# Anti-GAD Antibodies and the Cerebellum: Where Do We Stand**?**

**DOI:** 10.1007/s12311-018-0986-6

**Published:** 2018-10-20

**Authors:** Mario Manto, Hiroshi Mitoma, Christiane S. Hampe

**Affiliations:** 1 Service de Neurologie, Médiathèque Jean Jacquy, CHU-Charleroi, 6000 Charleroi, Belgium; 2 Service des Neurosciences, University of Mons, 7000 Mons, Belgium; 3 Medical Education Promotion Center, Tokyo Medical University, Tokyo, Japan; 4 University of Washington, School of Medicine, Seattle, WA 98109 USA

**Keywords:** Cerebellum, Cerebellar ataxia, Anti-GAD65 antibodies, Glutamic acid decarboxylase 65

## Abstract

Anti-GAD65 antibodies (anti-GAD65 Abs) are associated with cerebellar ataxia (CA). The significance of anti-GAD65 Abs has been a focus of debates. Since GAD65 is intracellularly located and associated with type 1 diabetes mellitus and different clinical neurological phenotypes such as CA, stiff-person syndrome, and epilepsy, some researchers have argued that anti-GAD65 Abs have no pathogenic roles. On the other hand, recent physiological studies in vitro and in vivo have elucidated that binding of GAD65 by anti-GAD65 Abs elicits loss of GAD65 functions pertaining GABA release with an epitope dependence, leading to the development of CA. Internalization of autoantibodies has been also clarified. These studies provide substantial evidence of the pathogenesis of anti-GAD65 Abs in CA. We also discuss methodological problems in the identification of anti-GAD65 Abs.

Conversion of glutamate to GABA is catalyzed by two isoforms of glutamate decarboxylase (GAD; GAD67 and GAD65). Autoantibodies to the smaller isoform (anti-GAD65 Abs) are associated with cerebellar ataxia (CA). The clinical entity of anti-GAD65 Ab-associated CA was established following the systematic survey of 14 patients by Honnorat et al. [1]. A recent large-scale survey of 1500 UK patients with progressive CAs [2] classified 2% of the patients with anti-GAD65 Ab-associated CA. The condition affects mostly women in their 50–60s (mean age, 58 years) and shows either subacute or chronic onset frequently associated with type 1 diabetes mellitus (T1DM) [1, 3, 4]. Almost all patients show posture and gait ataxia, whereas limb ataxia, dysarthria, and nystagmus are observed in about 60 to 70% of the patients [1, 3, 4]. The patients have characteristically high titers of anti-GAD65Abs in the serum (10 to 100-fold, compared with T1DM) and in the CSF [1, 3, 4]. Combinations of immunotherapies (e.g., one or the combination of corticosteroids, intravenous immunoglobulins (IVIg), plasmapheresis, immunosuppressive, and rituximab) have been recommended [5]. The present editorial tackles solved and unsolved questions on the underlying pathogenic mechanisms of anti-GAD65 Ab-associated CA.

## Do Anti-GAD65 Abs Play a Pathogenic Role in the Development of CAs?

Anti-GAD65 Abs are considered by some researchers to play no role in the pathogenesis of anti-GAD65 Ab-associated CA based on the following two arguments [6, 7]: (1) GAD65 is intracellularly located on the cytosolic side of the vesicles together with the vesicular GABA transporter VGAT [8], and (2) anti-GAD65 Abs are associated with T1DM and different clinical neurological phenotypes as present in CAs, stiff-person syndrome (SPS), and epilepsy [6]. However, accumulating physiological evidence both in vitro and in vivo clearly indicates that anti-GAD65 Abs impair cerebellar GABAergic synapses, leading to clinical manifestations of CAs. GABAergic synapses play major roles in the cerebellar circuitry.

The addition of CSF IgGs obtained from patients with anti-GAD65 Ab-associated CA to cerebellar slices is associated with a depression of GABA release [9, 10] and their intracerebellar administration elicit deficits in the cerebellar control of the motor cortex in vivo [11, 12]. Importantly, the IgGs-induced synaptic impairment is completely abolished by absorption of anti-GAD65Abs by recombinant GAD65 [13]. Furthermore, human monoclonal anti-GAD65Ab b78 mimics the pathogenic effects, similar to those induced by CSF IgGs in both in vitro and in vivo preparations [12, 14]. On the other hand, synaptic impairment is observed only in wild-type slices, but not in GAD65-KO slices, where inhibitory synaptic transmissions are mediated through a compensatory mechanism by GAD67 [14]. It has also been shown that binding of anti-GAD65 Ab b78 to GAD65 interferes with the association of GAD65 with the cytosolic face of GABA-containing synaptic vesicles [14], which results in impairment of GABA packaging into the vesicles and shuttling of vesicles to the release site on the synaptic cleft [14].

Taken together, these experimental studies show that binding of GAD65 by anti-GAD65Abs elicits loss of GAD65 functions pertaining GABA release, leading to the development of CAs as a result of the major role of GABA in the cerebellar circuitry.

### Internalization of Anti-GAD65 Abs

During the last 3 decades, several studies clearly have demonstrated that IgGs penetrate cerebellar neurons both in vitro [15, 16] and in vivo [17–19]. We have confirmed the internalization of human monoclonal anti-GAD65Ab b78 in cultured AF5 cells [14, 20], and we have observed b78 in CA1 interneurons and Purkinje neurons shortly after injection in the medial septum/diagonal band and ipsilateral interpositus nucleus, respectively [21]. However, unequivocal evidence about the internalization and the access route is currently missing. In this regard, some have argued that the antigen might be temporally exposed during exocytosis, providing a chance for binding with the antibodies [22, 23].

### Epitope-Dependent Pathogenic Actions of Anti-GAD65 Abs

The anti-GAD65Abs elicit their pathogenic actions in an epitope-specific fashion [12, 14], and only anti-GAD65Abs with a distinct epitope specificity characteristic for CA patients interfere with GABA release mechanisms, whereas anti-GAD65Abs epitope specificities associated with T1DM have no effects on GABAergic neurotransmission [12, 14]. Considering the abovementioned mechanism of anti-GAD65 Ab-mediated impairment of GABAergic signal transmission, namely through the interference with the association of GAD65 with GABAergic vesicles, it is very likely that only anti-GAD65 Abs with epitope specificities that prevent this association will cause changes in GABA release. Furthermore, differences in neurological features in conditions associated pathologically with anti-GAD65 Abs can be attributed to differences in epitope specificity; for example, anti-GAD65 Abs in CA interfere with GABA release, whereas anti-GAD65 Abs in SPS block the synthesis of GAD [12, 24].

Unfortunately, technical issues are often skipped in the discussion. The identification of disease-specific GAD65 Abs is complicated by the conformational nature of many of these epitopes. This characteristic limits the use of peptides and deletion mutants for epitope mapping. Instead, fusion proteins of GAD65 and its closely related isoform GAD67 have aided in the definition of two major epitope regions located in the middle and at the carboxy-terminal part of the molecule [25, 26]. However, even these fusion proteins do not always faithfully represent the three-dimensional structure of GAD65, resulting in loss of epitopes [27]. Competition assays with patients’ sera and recombinant Fab derived from GAD65-specific monoclonal antibodies have allowed the identification of specific conformational epitopes associated with disease [28, 29]. The use of these different epitope mapping methods likely explains the discrepancies in results pertaining to disease-specific anti GAD65 Ab epitopes [30–33].

## Does Heterogeneous Prognosis Suggest Divergence in Autoimmunity and What Is the Relationship with the Cerebellar Reserve?

The clinical course and the response to immunotherapies are heterogeneous. For example, some CA patients show good response to combination immunotherapy, and their symptoms improve to the extent of diminishing difficulties in daily lives [5], whereas the same immunotherapy does not provide benefit in other patients [5]. Our literature review article, in which 27 published clinical trials in 17 patients were reviewed [5], identified three factors that determine good prognosis: age less than 60 years, subacute clinical course, and absence of cerebellar atrophy.

After halting to disease progression, cerebellar lost function may be regained through the process of cerebellar restoration and compensation, which is termed cerebellar reserve [34]. Cerebellar reserve is preserved in younger patients and in patients free of cerebellar atrophy. It is critically important to consider the delay between symptoms onset and ataxia before concluding that therapies are ineffective.

Furthermore, the differences in prognosis between the subacute type and chronic type suggest a heterogeneous nature of autoimmunity. Cerebellar atrophy is observed in 75% of patients with the chronic type but is absent in the subacute type, suggesting that the immune responses in the chronic type progress to induce cell death resulting in clinical symptoms. The underlying mechanisms of this severe autoimmunity remains unclear. Impairment of immune tolerance might be involved as seen in paraneoplastic syndrome [35] or some autoimmune responses might coexist (for example, polyclonal anti-GAD65 Abs recognizing different epitopes or the dual actions of autoantibody- and cell-mediated mechanisms). The heterogeneous autoimmunity in the anti-GAD65 Ab-associated CA spectrum should be a focus of interest.

## Conclusion: the Need to Identify Prodromal Symptoms in Order to Preserve the Cerebellar Reserve

The aim of immunotherapies is to halt the disease progression before irreversible neuronal death in the cerebellum, that is cerebellar reserve, is lost. Time from symptoms onset to diagnosis is thus a key factor. We commonly encounter patients in whom the delay between symptoms onset and diagnosis extends beyond 2 years, impacting on cerebellar reserve. Efforts to the identification of prodromal symptoms might lead to very early therapies with good outcome [36]. Consensus studies on immune mechanisms are expected to define appropriate immunotherapies on a case-by-case scenario. The mechanisms reported in this editorial do not exclude at all the involvement of cell-mediated autoimmunity.
